# Ten Rules Governing the Administration of Ether

**Published:** 1895-12

**Authors:** Wm. B. Conway

**Affiliations:** Athens, Ga.


### TEN RULES GOVERNING THE ADMINISTRATION OF ETHER.

ATHENS, GA., November 17, 1895.

1. Place on a table near at hand a glass of water and one of brandy. A hypodermic syringe charged with nitrate of strychnia $\frac{1}{60}$ grain. A small bottle of aromatic spirits of ammonia, a tenaculum and a mouth gag, and one pound of Squibb's ether.

2. The stomach should be empty; clothes loose and light; a starched napkin, made into a cone, and a pledget of cotton or a handkerchief placed in the cone to receive the ether.

3. Allow no talking in the room. Admininister slowly for the first few minutes and then push the ether.

4. Watch carefully the pulse, respiration, and reflex of the eye, and if there are any signs of syncope, lower the head and stimulate the heart's action.

5. If any tendency to strangulation by the tongue falling against the larynx, fasten the tenaculum upon the tongue and pull it forward, allowing the tenaculum to remain on the tongue during the operation.

6. When the patient is thoroughly under the influence of the anesthetic remove the cone, and administer the ether from time to time, as sensibility returns.

7. Remove any kind of mucus about the mouth with the dressing forceps and a pledget of cotton. If any facial cyanosis occurs, remove the cone and induce artificial respiration.

8. If respiration becomes too rapid, stertorous or irregular, or the pulse very rapid and feeble, discontinue the ether at once.

9. If vomiting occurs, turn the patient upon one side so that the ejected matter may pass out without producing strangulation. So soon as the vomiting is over push the ether.

10. If any indication of heart failure, with pallor of the face, or weakening of all the forces, administer, at once, two or three syringefuls of dilute brandy or aromatic spirits of ammonia, alternating with nitrate of strychnia $\frac{1}{60}$ grain.

These are simple rules, but important to observe; and the cautious surgeon will not disregard them.

WM. B. CONWAY, M.D.

